# High Primary Production Contrasts with Intense Carbon Emission in a Eutrophic Tropical Reservoir

**DOI:** 10.3389/fmicb.2016.00717

**Published:** 2016-05-18

**Authors:** Rafael M. Almeida, Gabriel N. Nóbrega, Pedro C. Junger, Aline V. Figueiredo, Anízio S. Andrade, Caroline G. B. de Moura, Denise Tonetta, Ernandes S. Oliveira, Fabiana Araújo, Felipe Rust, Juan M. Piñeiro-Guerra, Jurandir R. Mendonça, Leonardo R. Medeiros, Lorena Pinheiro, Marcela Miranda, Mariana R. A. Costa, Michaela L. Melo, Regina L. G. Nobre, Thiago Benevides, Fábio Roland, Jeroen de Klein, Nathan O. Barros, Raquel Mendonça, Vanessa Becker, Vera L. M. Huszar, Sarian Kosten

**Affiliations:** ^1^Laboratory of Aquatic Ecology, Department of Biology, Instituto de Ciências Biológicas, Federal University of Juiz de ForaJuiz de Fora, Brazil; ^2^Departamento de Ciência do Solo, Escola Superior de Agricultura Luiz de Queiroz, University of São PauloPiracicaba, Brazil; ^3^Laboratory of Limnology, Federal University of Rio de JaneiroRio de Janeiro, Brazil; ^4^Laboratory of Water Resources and Environmental Sanitation, Federal University of Rio Grande do NorteNatal, Brazil; ^5^Laboratory of Limnology, Federal University of Rio Grande do NorteNatal, Brazil; ^6^Laboratory of Freshwater Ecology, Federal University of Santa CatarinaFlorianópolis, Brazil; ^7^Department of Aquatic Ecology and Environmental Biology, Institute for Water and Wetland Research, Radboud UniversityNijmegen, Netherlands; ^8^Departamento de Ecología Teórica y Aplicada, Centro Universitario Regional Este and Facultad de Ciencias, Universidad de la RepúblicaMontevideo, Uruguay; ^9^Departamento de Ciências Naturais, Universidade Federal do Estado do Rio de JaneiroRio de Janeiro, Brazil; ^10^Laboratory of Microbial Processes and Biodiversity, Federal University of São CarlosSão Carlos, Brazil; ^11^Aquatic Ecology and Environmental Sciences, Wageningen UniversityWageningen, Netherlands; ^12^Department of Ecology and Genetics, Uppsala UniversityUppsala, Sweden; ^13^Laboratório de Ficologia, Museu Nacional, Universidade Federal do Rio de JaneiroRio de Janeiro, Brazil

**Keywords:** carbon dioxide, methane, organic carbon burial, net ecosystem production, semiarid, Caatinga

## Abstract

Recent studies from temperate lakes indicate that eutrophic systems tend to emit less carbon dioxide (CO_2_) and bury more organic carbon (OC) than oligotrophic ones, rendering them CO_2_ sinks in some cases. However, the scarcity of data from tropical systems is critical for a complete understanding of the interplay between eutrophication and aquatic carbon (C) fluxes in warm waters. We test the hypothesis that a warm eutrophic system is a source of both CO_2_ and CH_4_ to the atmosphere, and that atmospheric emissions are larger than the burial of OC in sediments. This hypothesis was based on the following assumptions: (i) OC mineralization rates are high in warm water systems, so that water column CO_2_ production overrides the high C uptake by primary producers, and (ii) increasing trophic status creates favorable conditions for CH_4_ production. We measured water-air and sediment-water CO_2_ fluxes, CH_4_ diffusion, ebullition and oxidation, net ecosystem production (NEP) and sediment OC burial during the dry season in a eutrophic reservoir in the semiarid northeastern Brazil. The reservoir was stratified during daytime and mixed during nighttime. In spite of the high rates of primary production (4858 ± 934 mg C m^-2^ d^-1^), net heterotrophy was prevalent due to high ecosystem respiration (5209 ± 992 mg C m^-2^ d^-1^). Consequently, the reservoir was a source of atmospheric CO_2_ (518 ± 182 mg C m^-2^ d^-1^). In addition, the reservoir was a source of ebullitive (17 ± 10 mg C m^-2^ d^-1^) and diffusive CH_4_ (11 ± 6 mg C m^-2^ d^-1^). OC sedimentation was high (1162 mg C m^-2^ d^-1^), but our results suggest that the majority of it is mineralized to CO_2_ (722 ± 182 mg C m^-2^ d^-1^) rather than buried as OC (440 mg C m^-2^ d^-1^). Although temporally resolved data would render our findings more conclusive, our results suggest that despite being a primary production and OC burial hotspot, the tropical eutrophic system studied here was a stronger CO_2_ and CH_4_ source than a C sink, mainly because of high rates of OC mineralization in the water column and sediments.

## Introduction

Lakes and reservoirs occupy only a small fraction of the Earth surface, which contrasts with the large role they play in the global carbon (C) cycle (Cole et al., 2007; Tranvik et al., 2009). Because of large inputs of terrestrial inorganic and organic carbon (OC), lakes and reservoirs annually emit 0.1 and 0.3 Pg of C as methane (CH_4_) and carbon dioxide (CO_2_), respectively (Bastviken et al., 2011; Raymond et al., 2013). In artificial lakes, riverine emissions downstream of dams are also substantial (Guérin et al., 2006). Although lakes and reservoirs are globally important sources of C, they can simultaneously act as net C sinks since a great portion of the terrestrially derived OC is potentially buried in their sediments (Dean and Gorham, 1998; Downing et al., 2008). On a global scale, estimates suggest that lakes and reservoirs are 2–3 times larger C sources than sinks (Cole et al., 2007; Tranvik et al., 2009), yet this overall balance is still uncertain because studies on C burial are far more scarce than on C evasion (Mendonça et al., 2012).

Different factors contribute to the variability in C evasion from lakes and reservoirs. Globally, there is a significant latitudinal gradient in C fluxes from these systems (Kosten et al., 2010). Tropical systems are hotspots for C emissions and account for 34% of global inland water emissions (Raymond et al., 2013), and tropical reservoirs emit 3 and 5 times more CH_4_ and CO_2_ than non-tropical ones, respectively (Barros et al., 2011). In reservoirs, age is negatively correlated to C evasion (Abril et al., 2005; Barros et al., 2011). On a global scale, age together with latitude and OC inputs explains 40% of CO_2_ and 54% of CH_4_ emissions from hydroelectric reservoirs (Barros et al., 2011). For CO_2_ in particular, dissolved inorganic C loading originating from soil respiration also contributes to evasion from lakes (Weyhenmeyer et al., 2015). External inorganic input may even support CO_2_ emission from net autotrophic systems as demonstrated for several temperate systems (McDonald et al., 2013).

Size is also an important regulator of C cycling in lakes and reservoirs. The global area of lentic inland water ecosystems is dominated by millions of lakes and reservoirs smaller than 1 km^2^ (Downing et al., 2006), and these systems play a large role in global freshwater C cycle (Bastviken et al., 2004; Downing, 2010; McDonald et al., 2012; Read and Rose, 2013). Small systems are generally characterized by higher water temperatures together with larger amounts of OC that increase C processing rates (Downing, 2010; Read and Rose, 2013). Moreover, oxygen concentrations tend to be lower in small lakes and reservoirs than in larger ones, enhancing greenhouse-gas emissions – particularly CH_4_ – but also C burial (Downing, 2010). C sequestration in the sediments of these small systems can be as great as in terrestrial systems (forests and grasslands) and oceans (Downing, 2010). Thus, including small freshwater systems in global C budgets is an important challenge (Downing et al., 2006; Downing, 2010).

In addition to inter-lake variability, spatial variation within systems can also be substantial. This spatial variation is influenced by changes in river inflow and residence time, as well as by heterogeneity in primary production with, for instance, higher primary production in lacustrine zones of eutrophic systems (Pacheco et al., 2015). Ignoring spatial variation can result in up to 25% error in total system gas fluxes in large systems (Roland et al., 2010), and single-site measurements can overestimate OC burial rates by over 50% (Mackay et al., 2012; Mendonça et al., 2014).

Finally, the interaction between aquatic C fluxes and trophic state has been increasingly investigated. Although there is large variability in CO_2_ water-atmosphere fluxes in eutrophic lakes (Kortelainen et al., 2006; Sand-Jensen and Staehr, 2009; Balmer and Downing, 2011), recent studies in temperate systems revealed that when eutrophication increases, lakes and reservoirs tend to have less intense CO_2_ effluxes because of high CO_2_ uptake by primary production (Gu et al., 2011; Trolle et al., 2012). One study carried out in 100s of US agriculturally eutrophic lakes indicated that 60% of them are CO_2_-undersaturated (Balmer and Downing, 2011). Mass balances indicate that some lakes with extreme primary production become CO_2_ sinks because CO_2_ fixed by primary producers is buried in sediments as OC (Pacheco et al., 2013). Even though some temperate eutrophic systems may still function as atmospheric CO_2_ sources (Sand-Jensen and Staehr, 2009; Balmer and Downing, 2011; Knoll et al., 2013), the high rates of OC burial in sediments frequently exceed CO_2_ emissions (Knoll et al., 2013; Pacheco et al., 2013) and sometimes even the sum of CO_2_ and CH_4_ emissions (Sobek et al., 2012).

In tropical systems, the knowledge of the interplay between eutrophication and aquatic C fluxes is incipient and highly uncertain. Two studies from a Brazilian eutrophic reservoir indicate that it functions as a CO_2_ source during the rainy season and a CO_2_ sink during the dry season (Roland et al., 2010; Pacheco et al., 2015). The overall greenhouse-gas (GHG) balance of the system, however, remains unknown as OC burial and CH_4_ emissions were not measured. In tropical sediments, OC mineralization rates are more substantial than in temperate sediments because of warmer temperatures (Gudasz et al., 2010; Cardoso et al., 2014), which leads to lower OC burial efficiencies (Alin and Johnson, 2007; Mendonça et al., 2012) and a higher return of inorganic C to the water column. In addition, even if eutrophic systems are CO_2_ sinks, they can still be overall GHG sources because CH_4_ emissions might increase with increasing trophic status (Moss et al., 2011).

Here, we evaluated the carbon budget of a small eutrophic reservoir in Brazil’s semiarid region through an intensive fieldwork that embraced measurements of a large set of carbon flux pathways. We hypothesized that in spite of being eutrophic this reservoir is a source of both CO_2_ and CH_4_ to the atmosphere and that the emissions to the atmosphere would be larger than the burial of OC in its sediments. This hypothesis was based on the following assumptions: (i) OC mineralization rates are high in warm water systems, so that water column CO_2_ production rates override the high C uptake by primary producers, and (ii) increasing trophic status creates favorable conditions for CH_4_ production.

## Materials and Methods

### Study Site

**FIGURE 1:**
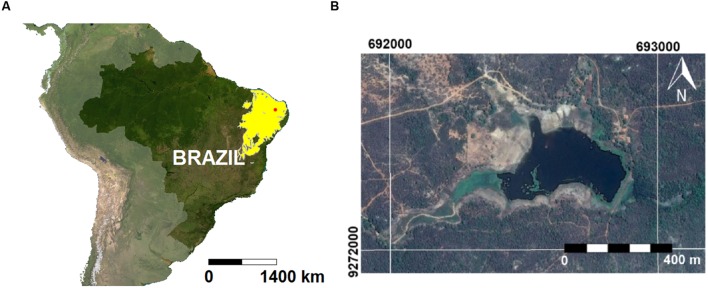
**(A)** Map of Brazil with emphasis to the Caatinga biome (yellow area) and the location of the ESEC reservoir (red dot). **(B)** Satellite image of the ESEC reservoir.

This study was developed in a small (0.2 km^2^; 160,000 m^3^), 70-years-old eutrophic water supply reservoir located partly inside the Ecological Station of Seridó (ESEC; 6°34′49″S; 37°15′20″W), a conservation unit of the semiarid Caatinga biome in northeastern Brazil (BSh climate, Köppen’s classification; **Figure 1**). The ESEC reservoir is part of the Piranhas-Assu watershed (44,600 km^2^), and it is used for irrigation, recreation and water supply for humans and animals. The riparian zone is used for agriculture, pasture and domestic activities. The land cover is dominated by xerophitic vegetation typical of the Caatinga biome. The region is characterized by moderately drained shallow soils (Chromic Luvisol), with high-activity clay and high base saturation at the clay-enriched subsurface soil horizon (IUSS Working Group WRB, 2014).

Brazilian semiarid reservoirs are largely under-studied considering that they are numerous and crucial to water supply. With an area of almost 1,000,000 km^2^, the Brazilian semiarid region is the most populated semiarid region on Earth (Barbosa et al., 2012). Accordingly, 100s of reservoirs have been constructed to compensate for the overall water deficit. In response to long water residence times, warm temperatures year-round and high loads of anthropogenic nutrients, Brazilian semiarid reservoirs are highly eutrophic (Lazzaro et al., 2003; Barbosa et al., 2012; Braga et al., 2015; Brasil et al., 2015). Eutrophication in these reservoirs is expected to become even more intense based on current climate change projections (Roland et al., 2012; Verspagen et al., 2014; Brasil et al., 2015). Our study was performed during the dry season, when eutrophication is generally maximum in Brazilian semiarid reservoirs because of high evaporation rates that make nutrients more concentrated (Barbosa et al., 2012; Braga et al., 2015).

**FIGURE 2:**
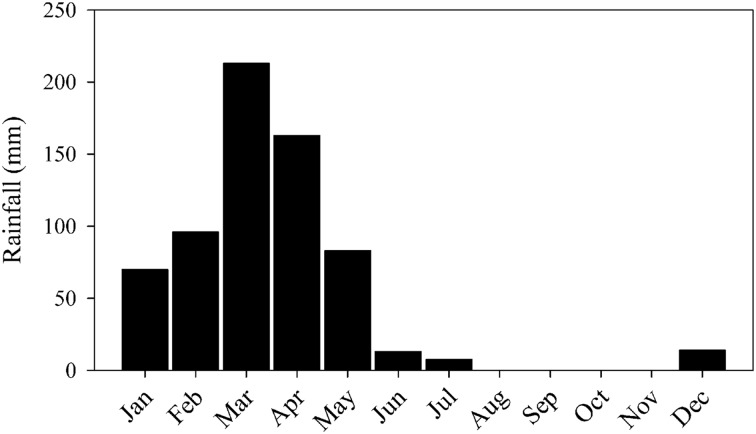
**Mean monthly rainfall between 1992 and 2014 in Serra Negra do Norte (Rio Grande do Norte, Brazil), near the ESEC reservoir.** Source: Rio Grande do Norte Agricultural Research Company (EMPARN).

The main source of water to the ESEC reservoir is precipitation. The regional climate is characterized as tropical semiarid, with low rainfall (∼700 mm per year) irregularly distributed throughout the year and an overall water deficit (Barbosa et al., 2012). Consequently, most rivers are temporary. Generally, the rainy season is concentrated in only 5 months (January to May), with practically no rainfall from July to November (**Figure 2**). The mean water depth during the sampling period (dry season) is 2 m, but the water column can be up to 4-m deep during the rainy season. During the dry season, the ESEC lake is highly eutrophic, with total phosphorus and chlorophyll-a concentrations of 98–104 and 31–64 μg L^-1^, respectively (Costa et al., 2015).

### Sampling and Analyses

**FIGURE 3:**
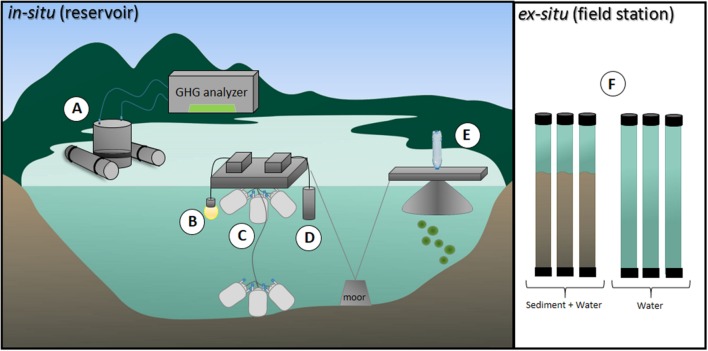
**Schematic representation of the field measurements made at the ESEC reservoir.** **(A)** CO_2_ and CH_4_ diffusive fluxes were determined through a floating chamber connected to a GHG analyzer. **(B)** A light meter was utilized to measure radiation 40 cm below at 15-min intervals. **(C)** CH_4_ oxidation was assessed as the CH_4_ concentration decline over time in closed and flexible airtight medical blood bags incubated at surface and bottom. **(D)** A multi-parameter water quality sonde was utilized to measure water temperature and dissolved oxygen (DO) 40 cm below surface at 15-min intervals. **(E)** CH_4_ ebullition was determined through bottom-moored funnels connected to water-filled glass bottles; the gas that accumulated in the bottles was measured in a GHG analyzer and CH_4_ was then calculated. **(F)** Sediment organic carbon (OC) mineralization was determined *ex situ* via dark incubations of cores containing sediment plus water and only water. Detailed information on the methods are shown in Section “Materials and Methods.”

Samples were taken during five consecutive days in July 2014, which characterizes the dry season (**Figure 2**). Dissolved oxygen (DO), water temperature, and radiation were measured every 15 min at 40 cm below surface at one location in the deepest part of the reservoir, with a luminescent sonde (LDO10115, Hach-Lange, Tiel, Netherlands), and a light meter, respectively (**Figure 3**).

Stratification and mixing patterns were assessed through temperature and DO vertical profiles performed using a luminescent sonde. Vertical profiles were measured in the morning and afternoon of July 15th, 16th, and 17th (with the exception of the morning of July 15th). Differences between the surface water temperature measured at 40 cm below the surface right before dawn and the bottom temperature measured during the day gave insight on the mixing pattern of the water column. Chlorophyll-a concentrations were measured at the surface and at the bottom at different sites between July 15th and 18th (*n* = 48) using a PHYTO-PAM (Heinz Walz GmbH, PHYTO-ED, Effelrich, Germany).

#### C Fluxes to the Atmosphere and CH_4_ Oxidation

Water-air diffusive CO_2_ and CH_4_ fluxes were measured in triplicate using transparent floating chambers (base area = 660 cm^2^; volume = 14,700 cm^3^) connected to a portable GHG analyzer that uses laser absorption spectroscopy (PICARRO G2508 GHG analyzer; **Figure 3**). The partial pressure of CO_2_, CH_4_ and N_2_O were measured continuously (every second) during 5-min intervals in the littoral (*n* = 8 for each gas) and pelagic zones (*n* = 6 for each gas), between 09:00 AM and 6:00 PM. The slope of the relationship between gas concentration and time was used to calculate the gas flux as follows:

$$F=\frac{V}{A}*slope*\frac{P*F1*F2}{R*T}$$

Where, F is gas flux (mg C m^-2^ d^-1^), V is chamber volume (m^3^), A is chamber surface area (m^2^), slope is the slope of the relationship between CH_4_ or CO_2_ and time (ppm/second); P is atmospheric pressure (kPa); F1 is the molecular weight for CO_2_ (44) or CH_4_ (16; g mole^-1^); F2 is the conversion factor of seconds to days; R is gas constant (8.3144 J K^-1^ mole^-1^); and T is temperature in Kelvin (K). Nitrous oxide (N_2_O) fluxes were also measured but remained below the detection limit. When fluxes from littoral and pelagic zones were significantly different (i.e., *t*-test, *p* < 0.05), the average flux from the reservoir was presented as a weighted average, which considers the area of both zones.

Water-air diffusive CO_2_ fluxes were also estimated from pH and alkalinity (Stumm and Morgan, 1996). pH was measured with a pH-meter (HPH-1002) and alkalinity was assessed through titration with 0.02 N sulfuric acid (*n* = 9). CO_2_ concentrations were then calculated using temperature-adjusted equilibrium constants. CO_2_ fluxes were calculated based on CO_2_ concentrations, CO_2_ saturation with the atmosphere and gas transfer velocity (k_600_ = 0.7 m d^-1^; Cole and Caraco, 1998), which was estimated based on wind records at the reservoir during the sampling days. Positive values denote CO_2_ efflux and negative values denote CO_2_ influx. Wind speed measurements were made 1.5 m above the water level and varied from 1.3 to 2.6 m s^-1^ (average = 1.8 m s^-1^). These wind speeds were normalized to wind speed at 10 m above surface (Smith, 1985).

To estimate CH_4_ ebullitive fluxes, we deployed bottom-moored triplicated funnels (base area = 800 cm^2^; volume = 1,060 cm^3^) connected to water-filled glass bottles at the pelagic zone of the reservoir during 24-h periods (*n* = 3; Rosa et al., 2003; **Figure 3**). Once ebullition occurs, the bubbles expel water from the bottles and form a headspace whose volume is equal to the bubble volume. Gas concentrations were determined in the Picarro G2508 GHG analyzer. To further calculate CH_4_ flux through ebullition, the gas volume that accumulated inside the water-filled bottles was calculated by adding water to the bubble-formed headspace using a graduated pipette.

Methane oxidation was assessed as the CH_4_ concentration decline over time in closed and flexible airtight medical blood bags (as in Bastviken et al., 2008; **Figure 3**). The 500 ml bags were filled with water from the surface (*n* = 9) and from the bottom (*n* = 8). Care was taken to prevent gas bubble formation in the bags. Initial samples for CH_4_ concentration (10 ml) were taken with a plastic syringe and transferred to evacuated and pre-capped 13-ml infusion vials. In order to preserve the samples, 25 μL of 2.45 M H_2_SO_4_ was added to the vials. The bags were incubated *in situ* at the same depth the water was sampled from (surface or near-bottom). Subsequent samples were taken from the bags after approximately 4 and 8 h. In the lab, 3 mL of atmospheric air was injected in each vial to create a headspace. After equilibration between the headspace and the water sample, the CH_4_ concentration in the headspace was measured with the PICARRO G2508 GHG analyzer.

#### Net Ecosystem Production

The 15-min interval measurements of DO concentrations were used to calculate the lake system respiration, gross primary production (GPP) and net ecosystem production (hereafter NEP-system) according to Cole et al. (2000). The change in DO (ΔDO) in each 15-min interval is due to NEP and diffusive O_2_ exchange (D) with the atmosphere (i.e., ΔDO = NEP + D). Diffusion (D) can be calculated using the measured concentration in the water, the concentration in the water in equilibrium with the atmosphere (O_2_-sat) and the gas transfer velocity (k), using the equation D = k(O_2_ - O_2_-sat). Hence, the NEP of each time interval can be calculated based on DO change and calculated O_2_ diffusion. Nighttime NEP (between 7 PM and 5 AM, which was the period the water column was mixed) was used to estimate R. One R was assigned to each day. Subsequently, we calculated GPP as the summation of NEP and R.

The vertical temperature and DO profiles showed that the DO sonde was continuously situated (at 40 cm below surface) in the upper mixing zone. Under mixed conditions, the NEP-system incorporates changes in DO caused by sediment respiration. By contrast, during stratification, oxygen-depleted waters resulting from hypolimnetic respiration accumulate in the bottom and are not diffused to the upper layers, so that change in DO is governed by planktonic metabolism. When the water column mixes at night, the sonde eventually captures sediment respiration that occurred during stratified periods. To obtain the pelagic NEP (hereafter NEP-pelagic, which results strictly from planktonic metabolism), we subtracted sediment respiration (i.e., sediment OC mineralization – see Materials and Methods, sub-heading “OC mineralization and burial in sediments”) from the NEP-system. Sediment primary production was assumed to be non-existent, as there is no sunlight reaching the sediment. The assessment of both NEP-system and NEP-pelagic allowed us to infer the contribution of planktonic and sediment respiration to the system respiration. Conversions of oxygen fluxes to CO_2_ fluxes were done assuming a respiratory quotient of 1:1.

#### OC Burial and Mineralization in Sediments

Organic carbon burial in sediments was assessed with sediment coring according to Mendonça et al. (2014). Sediment cores were sampled from nine different sites about evenly distributed using a gravity corer equipped with a hammer device (6 cm internal diameter and 120-cm long cores, UWITEC, Mondsee, Austria). The core was hammered into the sediment in order to retrieve cores containing the entire sediment layer at the sampling site, including some pre-flooding substrate. The transition between pre-flooding substrate and reservoir sediment was visually identified in the field. Sediment cores from the post-flooding substratum were sub-sampled in 2–6 cm thick slices that were stored refrigerated in airtight plastic containers until laboratory analysis. Dry sediment mass of each sediment slice was measured gravimetrically. OC content of all slices was determined in a C analyzer (Shimadzu, TOC-V CPN) coupled to a solid sample module (SSM 5000A). Because OC content in the three analyzed cores showed low variability (5.6 ± 0.8, average ± SD), we used the average OC content to calculate burial in the other six cores.

The OC burial rate calculations were, analogous to Mendonça et al. (2014), based on OC mass results. OC mass (g C) in each sediment slice was measured as the product of OC content (g g^-1^) and dry sediment mass (g). Total OC mass in each core was calculated as the sum of OC mass in all post-flooding sediment slices. Areal OC burial rates (g C m^-2^ year^-1^) for each core were calculated from total OC mass (g C), core surface area (2.8 × 10^-3^ m^2^) and the reservoir age (69 years in 2014). This results in a life-time average burial rate.

Integrated OC mineralization of the metabolically active layers of sediment was assessed as the change in DO resulting from aerobic processes in the sediment and the oxidation of anaerobically produced CH_4_. Sediment cores (6 cm internal diameter and 60 cm long) containing approximately 20 cm of sediment (i.e., the metabolically active layer of sediments; Burdige, 2007) collected from different locations in the pelagic zone were promptly incubated on land in a dark room (*n* = 5), at *in situ* temperature (26°C). For each incubation triplicates of cores with sediment plus lake water and cores containing only lake water from the same site were incubated gastight without headspace (**Figure 3F**). The cores with only lake water were used to assess DO decrease due to respiratory processes in the water, excluding the sediments. DO concentrations were measured right upon incubation and after 4 h in all cores – the average DO concentration before incubation was 6.9 mg L^-1^. The change in DO concentration through time multiplied by the water column height in each core resulted in DO consumption rates (i.e., respiration rates) per unit area. The areal respiration rates in the cores containing sediment subtracted by the areal respiration in cores containing only water reflected the OC mineralization in the sediment. DO concentrations did not reach hypoxia in any of the incubations (average final concentration was 6.1 mg L^-1^). Also, during our sampling campaign, stratification was never long enough for hypolimnetic DO concentrations to reach values below 4 mg/L, justifying our aerobic incubations. OC mineralization is probably underestimated as we likely missed anaerobic mineralization (i.e., likely not all anaerobically produced CH_4_ is oxidized in the overlying water in the core). Conversions of oxygen fluxes to CO_2_ fluxes were done assuming a respiratory quotient of 1:1.

Organic carbon burial efficiency, defined as the percentage of the total OC reaching the sediments that remains in the sediment (i.e., that escapes mineralization), was calculated as the ratio between OC burial rate and OC gross sedimentation rate (both in g C m^-2^ year^-1^). The OC gross sedimentation rate was calculated as the sum of OC burial and OC mineralization, considering that these are the two possible fates of OC that reaches the sediment.

## Results

### Mixing Pattern and Chlorophyll-a

**FIGURE 4:**
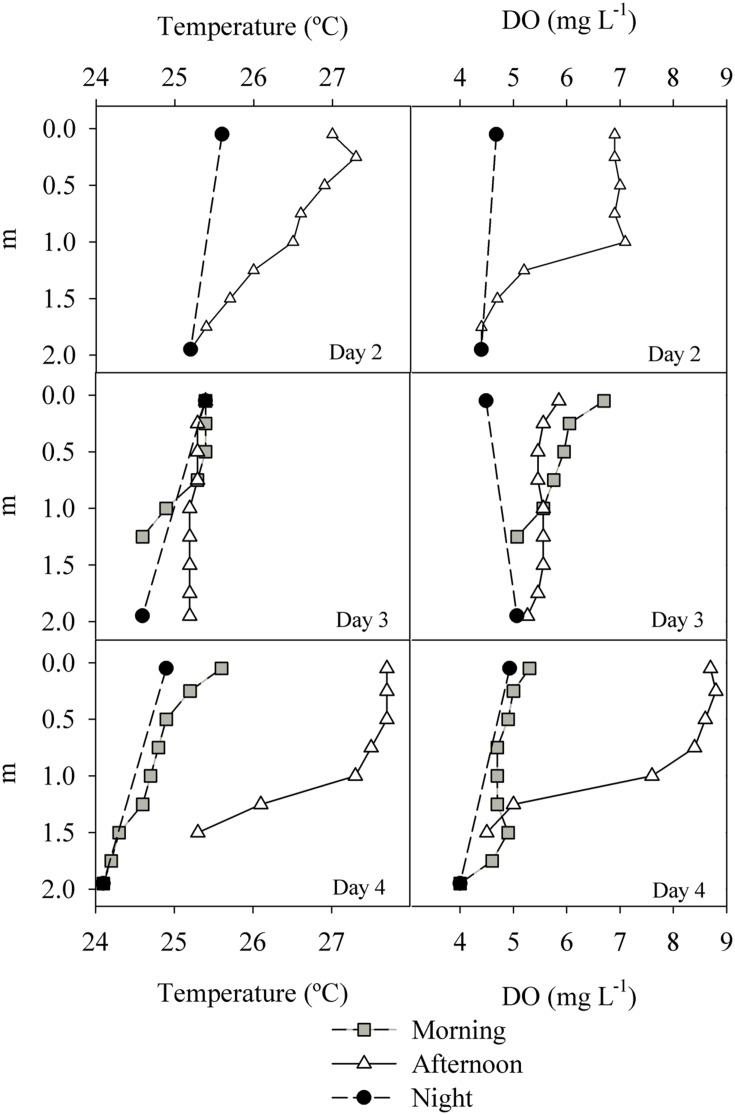
**Temperature and DO profiles measured in the ESEC reservoir between July 15th and 17th (“Day 2” and “Day 4”).** Night profiles refer to measurements taken right before dawn.

The temperature variation between surface and bottom waters was generally of 1–2°C as determined from vertical profiles, without any persistent thermocline. In the afternoon of the last day (July 17th), the water column stratified as indicated by both DO and temperature profiles (**Figure 4**). The vertical variation of DO accompanied the variation in temperature, with decreasing concentrations toward the bottom. Both temperature and DO profiles indicate that the water column fully mixes every night (**Figure 4**).

Chlorophyll-a concentrations showed little variability (51–69 μg L^-1^ considering surface and bottom samples together; average ± SD = 60 ± 5 μg L^-1^; data not shown). The concentrations of chlorophyll in illuminated epilimnetic waters were not significantly higher than in dark hypolimnetic waters (*t-*test; *p* > 0.05), reinforcing that the water column undergoes constant mixing.

### C Fluxes to the Atmosphere and CH_4_ Oxidation

**FIGURE 5:**
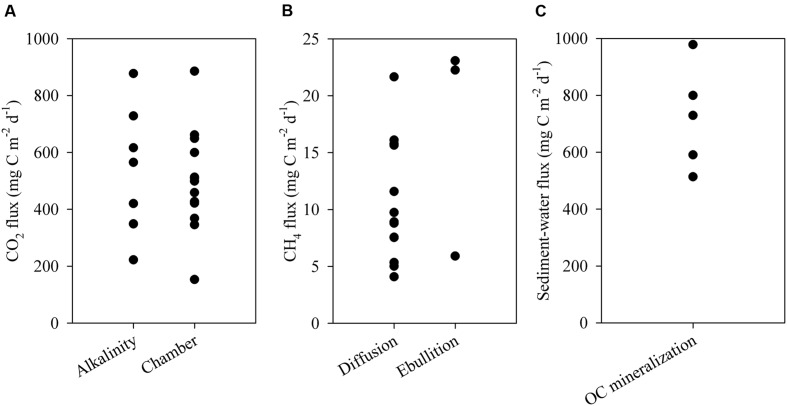
**(A)** Water-atmosphere fluxes of CO_2_ measured via alkalinity and floating chambers; **(B)** ebullitive and diffusive fluxes of CH_4_ to the atmosphere; **(C)** sediment OC mineralization measured *ex situ*. Each data point represents one measurement.

**FIGURE 6:**
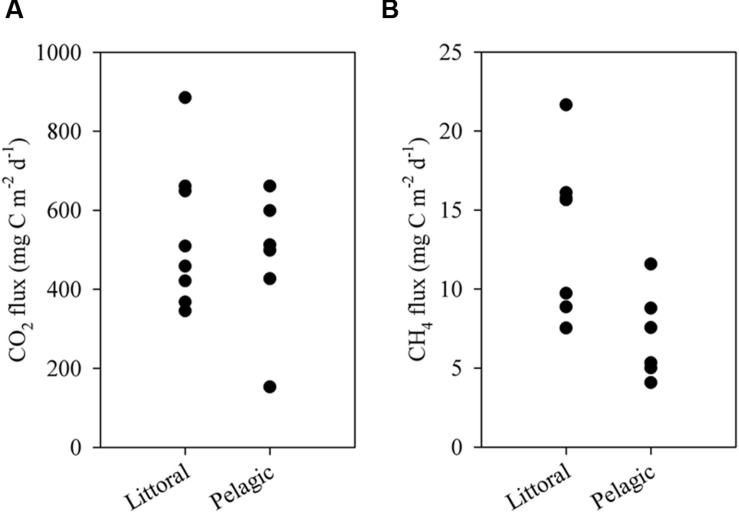
**Diffusive fluxes of CO_2_**(A)** and CH_4_**(B)** to the atmosphere in the littoral and pelagic zones of the ESEC reservoir.** Each data point represents one measurement.

The ESEC reservoir was a source of CO_2_ to the atmosphere as indicated by CO_2_ fluxes calculated directly via the floating chamber and indirectly through alkalinity and pH (**Figure 5**). The estimate from alkalinity and pH resulted in an average CO_2_ efflux of 497 ± 213 (SD) mg C m^-2^ d^-1^, whereas the direct measurement with the floating chamber resulted in an average flux of 518 ± 182 (SD) mg C m^-2^ d^-1^ (**Figure 5**). CO_2_ fluxes calculated based on alkalinity and directly measured in floating chambers did not significantly differ (*t*-test, *p* > 0.05). Combining the results of both methods, the average CO_2_ efflux from the ESEC reservoir was 510 mg C m^-2^ d^-1^. There was no significant spatial variation in CO_2_ efflux, with littoral and pelagic zones displaying similar fluxes (**Figure 6**).

The ESEC reservoir was also a source of CH_4_, which evades through ebullition (∼60%) and diffusion (∼40%). The average CH_4_ diffusive flux was 11 ± 6 mg C m^-2^ d^-1^ (average ± SD), whereas the CH_4_ ebullitive flux averaged 17 ± 10 mg C m^-2^ d^-1^ (average ± SD; **Figure 5**). There was significant spatial variation in CH_4_ diffusive fluxes, as the littoral zone diffused about two times more CH_4_ than the pelagic zone (**Figure 6**). The average CH_4_ diffusion, weighted by the area of the littoral and pelagic zones, was 11 mg C m^-2^ d^-1^. The summation of average ebullitive and diffusive fluxes results in a total CH_4_ efflux of 28 mg C m^-2^ d^-1^. The oxidation of CH_4_ averaged 31 ± 17 μg C m^-2^ d^-1^ at the surface and 18 ± 12 μg C m^-2^ d^-1^ at the bottom.

### OC Mineralization and Burial in Sediments

Sediment OC mineralization rates as determined from *ex situ* experiments were 722 ± 182 mg C m^-2^ d^-1^ (average ± SD; **Figure 5**). The post-flooding sediment layer varied in thickness from 24 to 62 cm, which translated into a maximum sediment deposition rate of 0.9 cm year^-1^ (average = 0.5 cm year^-1^). The mean OC content of post-flooding sediment averaged 5.6 ± 0.8% (±SD). Thus, the sediment OC stock averaged 10.4 ± 4.2 kg C m^-2^ (±SD). Based on the OC content in the sediment cores, the core area and the reservoir age, the OC burial rates varied from 88 to 280 g C m^-2^ year^-1^, averaging 161 g C m^-2^ year^-1^ (440 mg C m^-2^ d^-1^). Finally, considering the average rates of OC mineralization and burial, the OC sedimentation rates ranged from 307 to 499 g C m^-2^ year^-1^, averaging 380 g C m^-2^ year^-1^ (1040 mg C m^-2^ d^-1^). The OC sedimentation and burial rates resulted in a burial efficiency of 29 ± 5% (average ± SD).

### Net Ecosystem Production

**FIGURE 7:**
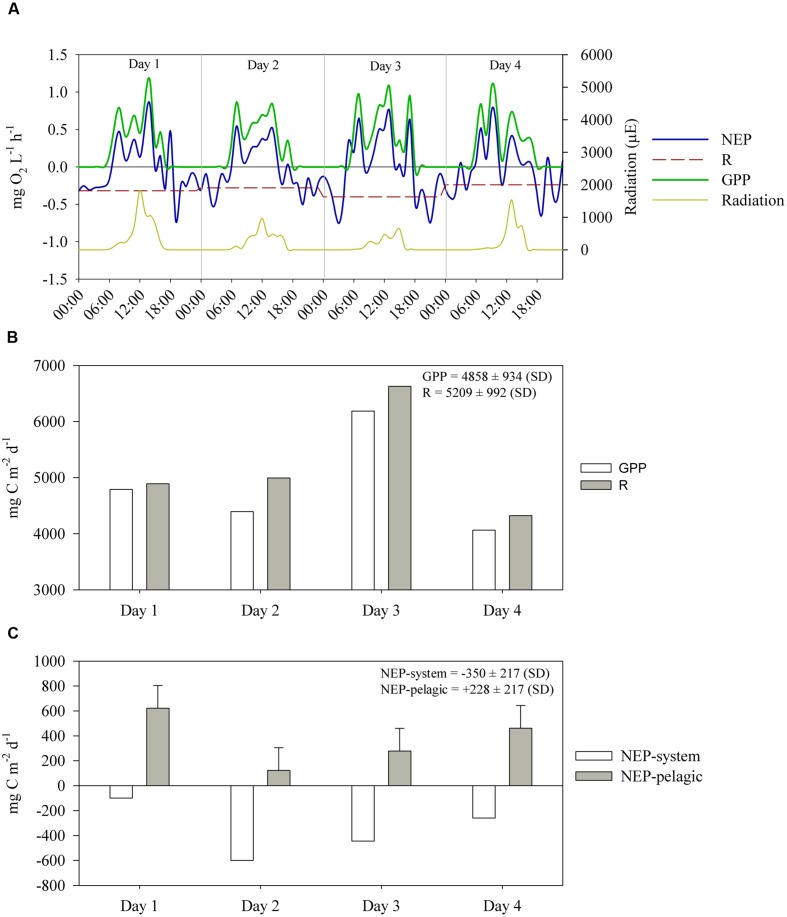
**(A)** Hourly estimates, in oxygen units, of volumetric gross primary production (GPP), respiration (R) and net ecosystem production (NEP), between July 14 and 17. R is shown as an average of NEP during nighttime on each day. The average R over the 4 days was 0.31 ± 0.07 mg O_2_ L^-1^ h^-1^. **(B)** Daily estimates, in carbon units, of GPP and R. **(C)** Daily estimates, in carbon units, of areal NEP-system (i.e., considering sediment respiration) and NEP-pelagic (i.e., excluding sediment respiration, 722 ± 182 mg C m^-2^ d^-1^) between July 14 and 17. The whiskers outside NEP-pelagic bars indicate the standard deviation of sediment respiration.

The diel curves of volumetric NEP, GPP and respiration indicated substantial variation in NEP (**Figure 7A**). Integration of NEP, GPP and R over 24 h indicated that although daytime measurements indicated net autotrophy (**Figure 7A**), the 24-h integrated NEP indicated that respiration (5209 ± 992 mg C m^-2^ d^-1^, average ± SD) exceeded GPP (4858 ± 934 mg C m^-2^ d^-1^, average ± SD) on the 4 days (**Figure 7B**); thus, the system was net heterotrophic throughout the sampling period (**Figure 7C**). On the other hand, when sediment respiration (i.e., OC mineralization in sediments, shown in see OC Mineralization and Burial in Sediments) was discounted to calculate the NEP of the pelagic system, we observed net autotrophy on all days (**Figure 7C**). Even if the highest sediment respiration rate estimated over the 4 days is considered (error bars in **Figure 7C**), the pelagic system still remains autotrophic, except on day 2. The average rates of NEP-system and NEP-pelagic were -350 ± 217 and 228 ± 217 mg C m^-2^ d^-1^, respectively. The most heterotrophic days were days 2 and 3 (**Figure 7C**), when light intensity was lower (**Figure 7A**); conversely, the least heterotrophic day was day 1, when light intensity was highest.

## Discussion

### Pelagic Metabolism and Water-Air C Fluxes

The strong variation in NEP (**Figure 7A**) is likely a reflection of the variability in irradiation and mixing of the water column, with a strong positive NEP in the early morning resulting from a combination of increased radiation and the onset of stratification. During stratification, the lower water layers become oxygen-depleted (**Figure 4**) which is reflected in the sonde-derived NEP estimates only when the water column mixes again (i.e., strong negative NEP after sunset; ∼18:00 h onward).

Overall, our findings indicate that the ESEC reservoir has an extremely high primary production (average GPP ∼4600 mg C m^-2^ d^-1^), which is corroborated by high concentrations of chlorophyll-a (average ∼60 μg L^-1^) and total phosphorus (∼100 μg L^-1^; Costa et al., 2015) classifying it as a hypertrophic system (Wetzel, 2001). The strong CO_2_ uptake due to the high GPP is counterbalanced by the extremely high ecosystem respiration. The measured respiration rates (∼5100 mg C m^-2^ d^-1^) are higher than the respiration of over 35 global ecosystem types ranging from 3 mg C m^-2^ d^-1^ (Arctic desert) to 4178 mg C m^-2^ d^-1^ (tropical agriculture soil; Doering et al., 2011, and references therein). As a result, during the sampling period (representative of the dry season), we found prevalence of positive NEP-pelagic and negative NEP-system. This suggests that if sediment respiration is disregarded, as in the case of NEP-pelagic, the system is prevalently autotrophic; on the contrary, if sediment respiration is taken into consideration, the system is prevalently heterotrophic. The heterotrophic nature of the system is corroborated by the prevalence of strong atmospheric CO_2_ efflux (**Figure 5A**).

Our estimate of the CO_2_ efflux from the ESEC reservoir likely represents the lower end of the daily and yearly variation in CO_2_ efflux, as our estimate is based on daytime effluxes measured during the dry season, a season that is likely to last longer and become drier over the coming years (Marengo et al., 2010). Aquatic primary production tends to peak during the dry season and loading of terrestrial C is minimal (Barbosa et al., 2012). Indeed, temporally resolved measurements in other Brazilian semiarid reservoirs (P. Junger, personal communication) and a hypertrophic tropical reservoir (Roland et al., 2010; Pacheco et al., 2015) suggest that the peak of heterotrophy occurs in the rainy season, when terrestrial C loading is high and primary production is lower.

Even with the likely underestimation, the CO_2_ efflux from the ESEC reservoir is more than three times higher than the rates reported for other eutrophic systems worldwide (**Table 1**), which is likely due the high organic matter loading to the system in the wet period combined with high temperatures enhancing respiration rates (Gudasz et al., 2010; Scofield et al., 2015). Input of external inorganic C coming from soil respiration may also contribute (McDonald et al., 2013; Weyhenmeyer et al., 2015). The high CO_2_ efflux is in accordance with the small size of the reservoir (Downing, 2010), but contradicts with the finding that old reservoirs (the ESEC reservoir is 70 years-old) tend to emit less CO_2_ (Barros et al., 2011). Overall, the ESEC reservoir’s CO_2_ efflux falls within the range reported for (less eutrophic) tropical lakes and tropical non-Amazonian reservoirs, and it is higher than the rates reported for all other types of systems (**Table 1**).

**Table 1:** Comparison of carbon fluxes and organic carbon burial (OCB) in the ESEC reservoir with those found in other aquatic systems worldwide.

Climatic zone	System	Flux (mg C m^2^ d^-^^1^)				OCB (mg C m^2^ d^-^^1^)	Reference
				
		CO_2_ Total	CH_4_ Diffusion	CH_4_ Ebullition	CH_4_ Total		
Tropical	*Tropical semiarid eutrophic reservoir*	*510*	*11*	*17*	*28*	*440*	*This study*
	Brazilian oligotrophic hydroelectric reservoirs	151	16	–	–	116	Roland et al., 2010; Ometto et al., 2013; Mendonça et al., 2014
	Brazilian eutrophic hydroelectric reservoir	-1.2	36	–	–	–	Ometto et al., 2013; Pacheco et al., 2015
	Amazonian hydroelectric reservoirs	1096	–	–	137	–	Barros et al., 2011
	Tropical non-Amazonian hydroelectric reservoirs	685	–	–	41	–	Barros et al., 2011
Temperate	US (Ohio) eutrophic reservoir	31	–	–	–	841	Knoll et al., 2013
	US (Iowa) eutrophic reservoirs	-17.5	–	–	–	47–405	Downing et al., 2008; Pacheco et al., 2013
	Swiss mesoeutrophic reservoir	66	11	90	101	3049	DelSontro et al., 2010; Sobek et al., 2012
	Temperate lakes	271	4	10	14	–	Bastviken et al., 2004; Marotta et al., 2009
	Temperate hydroelectric reservoirs	290	–	–	2	–	Barros et al., 2011
	German eutrophic lakes	67–480	3–144	–	–	–	Casper et al., 2000, 2009
Boreal	Boreal hydroelectric reservoirs	206	–	–	6.8	–	Barros et al., 2011
	Boreal lakes	279	–	–	4.9	–	Phelps et al., 1998; Karlsson, 2001


*Negative values indicate flux into the aquatic system.*

The CH_4_ efflux of the ESEC reservoir is similar to those reported for tropical non-Amazonian hydroelectric reservoirs, and higher than the average of temperate and boreal systems (**Table 1**). Ebullition was the most important CH_4_ emission pathway (∼60%), which concurs with what has been found in temperate and boreal small lakes (Bastviken et al., 2004). As opposed to CO_2_ fluxes, CH_4_ diffusive fluxes showed significant spatial variation (**Figure 6**), with higher fluxes in the littoral zone that is dominated by rooted macrophytes. In shallow lakes, higher CH_4_ emissions near the shore may be attributable to larger availability of plant-derived organic matter that indirectly fuels methanogenesis (Bastviken et al., 2010). Moreover, sediments in shallow zones are more exposed to wind-driven turbulence, which favors the release of CH_4_.

### Sediment OC Stock, Burial, and Mineralization

The C stock in the ESEC reservoir’s sediment is high considering the C-impoverishment of the watershed. The Caatinga biome is characterized by shallow soils with xerophytic vegetation (Menezes et al., 2012). The C stock calculated for the ESEC reservoir (average = 10.4 kg C m^-2^) is about five times higher than the soil C stock for typical semiarid phytoecological units (2.0–3.1 kg C m^-2^; Nóbrega, 2013) and similar to the C stock in tropical semiarid mangroves (Nóbrega et al., 2016). Mangroves are among the most important C burial hotspots worldwide (Howard et al., 2014), which suggests that the sediment of Brazilian semiarid reservoirs may act as important regional C sinks.

The OC burial in the ESEC reservoir (161 g m^-2^ year^-1^) is higher than the values reported for temperate oligotrophic lakes (**Table 1**). Indeed, OC burial rates are commonly higher in eutrophic systems due to the fast deposition of highly organic sediments (Downing et al., 2008). The OC burial efficiency in the ESEC reservoir (average = 29%) fits the range of temperate lake sediments primarily composed by autochthonous OC (3–50%; Sobek et al., 2009). Data on OC burial efficiency in tropical systems that we know of is limited to one hydroelectric reservoir (Lake Kariba, 41%, Kunz et al., 2011) and one natural lake (Lake Kivu, ∼50%, Sobek et al., 2009), all showing higher efficiencies than the ESEC reservoir. The higher OC burial efficiencies in the hydroelectric reservoirs are due to the high sediment load from inflowing rivers while Lake Kivu sediments bury carbon efficiently because it is permanently anoxic.

The ESEC reservoir sediment OC mineralization is high and falls within the upper range of rates reported for worldwide lakes (Gudasz et al., 2010; Cardoso et al., 2014). This is probably because in the ESEC reservoir bottom waters are frequently oxygenated, temperatures are consistently high and the water column is shallow (Sobek et al., 2009; Gudasz et al., 2010). Overall, about 70% of the OC reaching the ESEC reservoir’s sediment is mineralized rather than buried. The typical labile properties of organic matter in eutrophic systems result in a low share of the sedimentary OC escaping mineralization (Sobek et al., 2009; Gudasz et al., 2010). In addition, high ambient temperatures also favor mineralization over burial (Gudasz et al., 2010; Marotta et al., 2014). The high percentage of OC mineralization is an important driver of the prevalent heterotrophic state of the reservoir and the subsequent atmospheric CO_2_ efflux observed (Kortelainen et al., 2006; Cardoso et al., 2013).

## Conclusion

Although our results demonstrate that the ESEC reservoir is a regional C burial hotspot, the sum of CO_2_ (510 mg C m^-2^ d^-1^) and CH_4_ (27 mg C m^-2^ d^-1^) emissions measured during the dry season outweighs the amount of OC burial in sediments (440 mg C m^-2^ d^-1^) in terms of C units. This outweighing becomes much more imbalanced toward C emissions if it is considered that CH_4_ is a GHG 34 times more potent than CO_2_ in a 100-years time interval (Myhre et al., 2013). Accounting for the global warming potential of CH_4_, we estimate that the ESEC reservoir emits about three times more CO_2_-equivalents to the atmosphere than it buries as OC in the sediment. Assuming that the reservoir was less heterotrophic at the time of sampling (i.e., in the dry season) than during the wet season, our estimate likely represents the lower end of yearly variation in CO_2_ efflux. Therefore, although temporally resolved data would render our findings more conclusive, our results suggest that the eutrophic ESEC reservoir is a much larger C source than it is a C sink. This is not in line with data from eutrophic and mesoeutrophic temperate lakes suggesting that they are larger C sinks than CO_2_ (Knoll et al., 2013; Pacheco et al., 2013) and C (i.e., CO_2_ plus CH_4_) sources (Sobek et al., 2012). In the ESEC reservoir, CH_4_ is responsible for the largest share of CO_2_-equivalents emissions, but even if CH_4_ emissions are disregarded, the system is still a slightly larger CO_2_ source than a C sink. Hence, the idea of eutrophication shifting lakes into CO_2_ sinks, as suggested for temperate eutrophic systems (Pacheco et al., 2013), may not be applicable to tropical eutrophic systems such as the semiarid, shallow ESEC reservoir. The underlying cause is probably the fact that temperate eutrophic systems bury a great deal of the C fixed via primary production, whereas warm eutrophic systems such as the ESEC reservoir respire a large share of this OC.

## Author Contributions

All authors listed participated in the fieldwork and have made substantial direct and intellectual contribution to the work, and approved it for publication.

## Conflict of Interest Statement

The authors declare that the research was conducted in the absence of any commercial or financial relationships that could be construed as a potential conflict of interest.

## References

[B1] Abril G. Guérin F. Richard S. Delmas R. Galy-Lacaux C. Gosse P. (2005). Carbon dioxide and methane emissions and the carbon budget of a 10-year old tropical reservoir (Petit Saut, French Guiana). *Global Biogeochem. Cycles* 19:GB4007 10.1029/2005GB002457

[B2] Alin S. R. Johnson T. C. (2007). Carbon cycling in large lakes of the world: a synthesis of production, burial, and lake-atmosphere exchange estimates. *Global Biogeochem. Cycles* 21:GB3002 10.1029/2006GB002881

[B3] Balmer M. B. Downing J. A. (2011). Carbon dioxide concentrations in eutrophic lakes: undersaturation implies atmospheric uptake. *Inland Waters* 1 125–132. 10.5268/IW-1.2.366

[B4] Barbosa J. E. D. L. Medeiros E. S. F. Brasil J. Cordeiro R. D. S. Crispim M. C. B. Silva G. H. G. D. (2012). Aquatic systems in semi-arid Brazil: limnology and management. *Acta Limnologica Brasiliensia* 24 103–118. 10.1590/S2179-975X2012005000030

[B5] Barros N. Cole J. J. Tranvik L. J. Prairie Y. T. Bastviken D. Huszar V. L. M. (2011). Carbon emission from hydroelectric reservoirs linked to reservoir age and latitude. *Nat. Geosci.* 4 593–596. 10.1038/NGEO1211

[B6] Bastviken D. Cole J. Pace M. Tranvik L. (2004). Methane emissions from lakes: dependence of lake characteristics, two regional assessments, and a global estimate. *Global Biogeochem. Cycles* 18:GB4009 10.1029/2004GB002238

[B7] Bastviken D. Cole J. J. Pace M. L. Van De Bogert M. C. (2008). Fates of methane from different lake habitats: connecting whole-lake budgets and CH4 emissions. *J. Geophys. Res. Biogeosci.* 113:G02024 10.1029/2007JG000608

[B8] Bastviken D. Santoro A. L. Marotta H. Pinho L. Q. Calheiros D. F. Crill P. (2010). Methane emissions from Pantanal, South America, during the low water season: toward more comprehensive sampling. environ. *Sci. Technol.* 44 5450–5455. 10.1021/es1005048 20568738

[B9] Bastviken D. Tranvik L. J. Downing J. A. Crill P. M. Enrich-Prast A. (2011). Freshwater methane emissions offset the continental carbon sink. *Science* 331:50 10.1126/science.1196808 21212349

[B10] Braga G. G. Becker V. Oliveira J. N. P. Mendonça J. R. Jr. Bezerra A. F. M. Torres L. M. (2015). Influence of extended drought on water quality in tropical reservoirs in a semiarid region. *Acta Limnologica Brasiliensia* 27 15–23. 10.1590/S2179-975X2214

[B11] Brasil J. Attayde J. Vasconcelos F. Dantas D. F. Huszar V. M. (2015). Drought-induced water-level reduction favors cyanobacteria blooms in tropical shallow lakes. *Hydrobiologia* 770 145–164. 10.1007/s10750-015-2578-5

[B12] Burdige D. J. (2007). Preservation of organic matter in marine sediments: controls, mechanisms, and an imbalance in sediment organic carbon budgets? *Chem. Rev.* 107 467–485. 10.1021/cr050347q 17249736

[B13] Cardoso S. J. Enrich-Prast A. Pace M. L. Roland F. (2014). Do models of organic carbon mineralization extrapolate to warmer tropical sediments? *Limnol. Oceanogr.* 59 48–54. 10.4319/lo.2014.59.1.0048

[B14] Cardoso S. J. Vidal L. O. Mendonça R. F. Tranvik L. J. Sobek S. Roland F. (2013). Spatial variation of sediment mineralization supports differential CO2 emissions from a tropical hydroelectric reservoir. *Front. Microbiol.* 4:101 10.3389/fmicb.2013.00101 PMC3639384 23641239

[B15] Casper P. Albino M. F. Adams D. D. (2009). Diffusive fluxes of CH4 and CO2 across the water-air interface in the eutrophic lake Dagow, northeast Germany. *Verh. Internat. Verein. Limnol.* 30 874–877.

[B16] Casper P. Maberly S. Hall G. Finlay B. (2000). Fluxes of methane and carbon dioxide from a small productive lake to the atmosphere. *Biogeochemistry* 49 1–19. 10.1023/A:1006269900174

[B17] Cole J. J. Caraco N. F. (1998). Atmospheric exchange of carbon dioxide in a low-wind oligotrophic lake measured by the addition of SF6. *Limnol. Oceanogr.* 43 647–656. 10.4319/lo.1998.43.4.0647

[B18] Cole J. J. Pace M. L. Carpenter S. R. Kitchell J. F. (2000). Persistence of net heterotrophy in lakes during nutrient addition and food web manipulations. *Limnol. Oceanogr.* 45 1718–1730. 10.4319/lo.2000.45.8.1718

[B19] Cole J. J. Prairie Y. T. Caraco N. F. Mcdowell W. H. Tranvik L. J. Striegl R. G. (2007). Plumbing the global carbon cycle: integrating inland waters into the terrestrial carbon budget. *Ecosystems* 10 171–184. 10.1007/s10021-006-9013-8

[B20] Costa M. R. A. Attayde J. L. Becker V. (2015). Effects of water level reduction on the dynamics of phytoplankton functional groups in tropical semi-arid shallow lakes. *Hydrobiologia* 1–15. 10.1007/s10750-015-2593-6

[B21] Dean W. E. Gorham E. (1998). Magnitude and significance of carbon burial in lakes, reservoirs, and peatlands. *Geology* 26 535–538. 10.1130/0091-7613

[B22] DelSontro T. Mcginnis D. F. Sobek S. Ostrovsky I. Wehrli B. (2010). Extreme methane emissions from a Swiss hydropower reservoir: contribution from bubbling sediments. *Environ. Sci. Technol.* 44 2419–2425. 10.1021/es9031369 20218543

[B23] Doering M. Uehlinger U. Ackermann T. Woodtli M. Tockner K. (2011). Spatiotemporal heterogeneity of soil and sediment respiration in a river-floodplain mosaic (Tagliamento, NE Italy). *Freshw. Biol.* 56 1297–1311. 10.1111/j.1365-2427.2010.02569.x

[B24] Downing J. A. (2010). Emerging global role of small lakes and ponds: little things mean a lot. *Limnetica* 29 9–23.

[B25] Downing J. A. Cole J. Middelburg J. J. Striegl R. G. Duarte C. M. Kortelainen P. (2008). Sediment organic carbon burial in agriculturally eutrophic impoundments over the last century. *Global Biogeochem. Cycles* 22:10 10.1029/2006GB002854

[B26] Downing J. A. Prairie Y. T. Cole J. J. Duarte C. M. Tranvik L. J. Striegl R. G. (2006). The global abundance and size distribution of lakes, ponds, and impoundments. *Limnol. Oceanogr.* 51 2388–2397. 10.4319/lo.2006.51.5.2388

[B27] Gu B. H. Schelske C. L. Coveney M. F. (2011). Low carbon dioxide partial pressure in a productive subtropical lake. *Aquat. Sci.* 73 317–330. 10.1007/s00027-010-0179-y

[B28] Gudasz C. Bastviken D. Steger K. Premke K. Sobek S. Tranvik L. J. (2010). Temperature-controlled organic carbon mineralization in lake sediments. *Nature* 466 1134–1134. 10.1038/nature09186 20651689

[B29] Guérin F. Abril G. Richard S. Burban B. Reynouard C. Seyler P. (2006). Methane and carbon dioxide emissions from tropical reservoirs: significance of downstream rivers. *Geophys. Res. Lett.* 33:L21407 10.1029/2006GL027929

[B30] Howard J. Hoyt S. Isensee K. Telszewski M. Pidgeon E. (eds) (2014). *Coastal Blue Carbon: Methods for Assessing Carbon Stocks and Emissions Factors in Mangroves, Tidal Salt Marshes, and Seagrasses.* Arlington, VA: Conservation International, Intergovernmental Oceanographic Commission of UNESCO, International Union for Conservation of Nature.

[B31] IUSS Working Group WRB (2014). *World Reference Base for Soil Resources. International Soil Classification System for Naming Soils and Creating Legends for Soil Maps.* World Soil Resources Reports No. 106 Rome: FAO.

[B32] Karlsson J. (2001). *Pelagic Energy Mobilization and Carbon Dioxide Balance in Subartic Lakes in Northern Sweden.* Ph.D. thesis, Umeå University, Umeå.

[B33] Knoll L. B. Vanni M. J. Renwick W. H. Dittman E. K. Gephart J. A. (2013). Temperate reservoirs are large carbon sinks and small CO2 sources: results from high-resolution carbon budgets. *Global Biogeochem. Cycles* 27 52–64. 10.1002/gbc.20020

[B34] Kortelainen P. Rantakari M. Huttunen J. T. Mattsson T. Alm J. Juutinen S. (2006). Sediment respiration and lake trophic state are important predictors of large CO2 evasion from small boreal lakes. *Global Change Biol.* 12 1554–1567. 10.1111/j.1365-2486.2006.01167.x

[B35] Kosten S. Roland D. Marques E. H. Van Nes N. Mazzeo L. D. L. Sternberg M. (2010). Climate-dependent CO2 emissions from lakes. *Global Biogeochem. Cycles* 24:GB2007 10.1029/2009GB003618

[B36] Kunz M. J. Wüest A. Wehrli B. Landert J. Senn D. B. (2011). Impact of a large tropical reservoir on riverine transport of sediment, carbon, and nutrients to downstream wetlands. *Water Resour. Res.* 47 W12531 10.1029/2011WR010996

[B37] Lazzaro X. Bouvy M. Ribeiro-Filho R. A. Oliviera V. S. Sales L. T. Vasconcelos A. R. M. (2003). Do fish regulate phytoplankton in shallow eutrophic Northeast Brazilian reservoirs? *Freshw. Biol.* 48 649–668. 10.1046/j.1365-2427.2003.01037.x

[B38] Mackay E. B. I Jones D. Folkard A. M. Barker P. (2012). Contribution of sediment focussing to heterogeneity of organic carbon and phosphorus burial in small lakes. *Freshw. Biol.* 57 290–304. 10.1111/j.1365-2427.2011.02616.x

[B39] Marengo J. A. Ambrizzi T. Da Rocha R. P. Alves L. M. Cuadra S. V. Valverde M. C. (2010). Future change of climate in South America in the late twenty-first century: intercomparison of scenarios from three regional climate models. *Clim. Dyn.* 35 1089–1113. 10.1007/s00382-009-0721-6

[B40] Marotta H. Duarte C. M. Sobek S. Enrich-Prast A. (2009). Large CO2 disequilibria in tropical lakes. *Global Biogeochem. Cycles* 23:GB4022 10.1029/2008GB003434

[B41] Marotta H. Pinho L. Gudasz C. Bastviken D. Tranvik L. J. Enrich-Prast A. (2014). Greenhouse gas production in low-latitude lake sediments responds strongly to warming. *Nat. Clim. Chang.* 4 467–470. 10.1038/NCLIMATE2222

[B42] McDonald C. P. Rover J. A. Stets E. G. Striegl R. G. (2012). The regional abundance and size distribution of lakes and reservoirs in the United States and implications for estimates of global lake extent. *Limnol. Oceanogr.* 57 597–606. 10.4319/lo.2012.57.2.0597

[B43] McDonald C. P. Stets E. G. Striegl R. G. Butman D. (2013). Inorganic carbon loading as a primary driver of dissolved carbon dioxide concentrations in the lakes and reservoirs of the contiguous United States. *Global Biogeochem. Cycles* 27 285–295. 10.1002/gbc.20032

[B44] Melo M. L. Almeida R. M. (2014). A report on course on metabolism of Brazilian Semiarid inland waters 14-20 July, 2014 (Ecological Station, Seridó (ESS), Brazil). *SIL News* 65 6–8.

[B45] Mendonça R. Kosten S. Sobek S. Barros N. Cole J. Tranvik L. (2012). Hydroelectric carbon sequestration. *Nat. Geosci.* 5 838–840. 10.1038/ngeo1653

[B46] Mendonça R. Kosten S. Sobek S. Cole J. J. Bastos A. C. Albuquerque A. L. (2014). Carbon sequestration in a large hydroelectric reservoir: an integrative seismic approach. *Ecosystems* 17 430–441. 10.1007/s10021-013-9735-3

[B47] Menezes R. S. C. Sampaio E. V. S. B. Giongo V. Perez-Marin A. M. (2012). Biogeochemical cycling in terrestrial ecosystems of the Caatinga Biome. *Braz. J. Biol.* 72 643–653. 10.1590/S1519-69842012000400004 23011295

[B48] Moss B. Kosten S. Meerhoff M. Battarbee R. W. Jeppesen E. Mazzeo N. (2011). Allied attack: climate change and eutrophication. *Inland Waters* 1 101–105. 10.5268/IW-1.2.359

[B49] Myhre G. Shindell D. Bréon F.-M. Collins W. Fuglestvedt J. Huang J. (2013). “Anthropogenic and natural radiative forcing,” in *Climate Change 2013: The Physical Science Basis. Contribution of Working Group I to the Fifth Assessment Report of the Intergovernmental Panel on Climate Change*, eds Stocker T. F. Qin D. Plattner G. K. Tignor M. Allen S. K. Boschung J. (New York, NY: Cambridge University Press).

[B50] Nóbrega G. N. (2013). *Blue Carbon in Semi-Arid Mangrove Soils: Importance, Quantification Methods and C-CO2 Gas Emissions.* Master’s thesis, Universidade Federal do Ceará, Fortaleza.

[B51] Nóbrega G. N. Ferreira T. O. Siqueira Neto M. Queiroz H. M. Artur A. G. Mendonça E. D. (2016). Edaphic factors controlling summer (rainy season) greenhouse gas emission (CO2 and CH4) from semiarid mangrove soils (NE-Brazil). *Sci. Total Environ.* 542 685–693. 10.1016/j.scitotenv.2015.10.108 26546764

[B52] Ometto J. P. Cimbleris A. C. P. Dos Santos M. A. Rosa L. P. Abe D. Tundisi J. G. (2013). Carbon emission as a function of energy generation in hydroelectric reservoirs in Brazilian dry tropical biome. *Energy Policy* 58 109–116. 10.1016/j.enpol.2013.02.041

[B53] Pacheco F. S. Roland F. Downing J. A. (2013). Eutrophication reverses whole-lake carbon budgets. *Inland Waters* 4 41–48. 10.5268/IW-4.1.614

[B54] Pacheco F. S. Soares M. C. S. Assireu A. T. Curtarelli M. P. Roland F. Abril G. (2015). The effects of river inflow and retention time on the spatial heterogeneity of chlorophyll and water–air CO2 fluxes in a tropical hydropower reservoir. *Biogeosciences* 12 147–162. 10.5194/bg-12-147-2015

[B55] Phelps A. R. Peterson K. M. Jeffries M. O. (1998). Methane efflux from high-latitude lakes during spring ice melt. *J. Geophys. Res. Atmos.* 103 29029–29036. 10.1029/98JD00044

[B56] Raymond P. A. Hartmann J. Lauerwald R. Sobek S. Mcdonald C. Hoover M. (2013). Global carbon dioxide emissions from inland waters. *Nature* 503 355–359. 10.1038/nature12760 24256802

[B57] Read J. S. Rose K. C. (2013). Physical responses of small temperate lakes to variation in dissolved organic carbon concentrations. *Limnol. Oceanogr.* 58 921–931. 10.4319/lo.2013.58.3.0921

[B58] Roland F. Huszar V. L. M. Farjalla V. F. Enrich-Prast A. Amado A. M. Ometto J. P. H. B. (2012). Climate change in Brazil: perspective on the biogeochemistry of inland waters. *Braz. J. Biol.* 72 709–722. 10.1590/S1519-69842012000400009 23011300

[B59] Roland F. Vidal L. O. Pacheco F. S. Barros N. O. Assireu A. Ometto J. P. H. B. (2010). Variability of carbon dioxide flux from tropical (Cerrado) hydroelectric reservoirs. *Aquat. Sci.* 72 283–293. 10.1007/s00027-010-0140-0

[B60] Rosa L. P. Dos Santos M. A. Matvienko B. Sikar E. Lourenco R. S. M. Menezes C. F. (2003). Biogenic gas production from major Amazon reservoirs, Brazil. *Hydrol. Process.* 17 1443–1450. 10.1002/hyp.1295

[B61] Sand-Jensen K. Staehr P. (2009). Net heterotrophy in small Danish lakes: a widespread feature over gradients in trophic status and land cover. *Ecosystems* 12 336–348. 10.1007/s10021-008-9226-0

[B62] Scofield V. Jacques S. M. S. Guimaraes J. R. D. Farjalla V. F. (2015). Potential changes in bacterial metabolism associated with increased water temperature and nutrient inputs in tropical humic lagoons. *Front. Microbiol.* 6:310 10.3389/fmicb.2015.00310 PMC4397971 25926827

[B63] Smith S. V. (1985). Physical, chemical and biological characteristics of CO2 gas flux across the air water interface. *Plant Cell Environ.* 8 387–398.

[B64] Sobek S. Delsontro T. Wongfun N. Wehrli B. (2012). Extreme organic carbon burial fuels intense methane bubbling in a temperate reservoir. *Geophys. Res. Lett.* 39 L01401 10.1029/2011GL050144

[B65] Sobek S. Durisch-Kaiser E. Zurbrugg R. Wongfun N. Wessels M. Pasche N. (2009). Organic carbon burial efficiency in lake sediments controlled by oxygen exposure time and sediment source. *Limnol. Oceanogr.* 54 2243–2254. 10.4319/lo.2009.54.6.2243

[B66] Stumm W. Morgan J. J. (1996). *Aquatic Chemistry: Chemical Equilibria and Rates in Natural Waters.* New York, NY: John Wiley and Sons, Inc.

[B67] Tranvik L. J. Downing J. A. Cotner J. B. Loiselle S. A. Striegl R. G. Ballatore T. J. (2009). Lakes and reservoirs as regulators of carbon cycling and climate. *Limnol. Oceanogr.* 54 2298–2314. 10.4319/lo.2009.54.6_part_2.2298

[B68] Trolle D. Staehr P. Davidson T. Bjerring R. Lauridsen T. Søndergaard M. (2012). Seasonal dynamics of CO2 flux across the surface of shallow temperate lakes. *Ecosystems* 15 336–347. 10.1007/s10021-011-9513-z

[B69] Verspagen J. M. H. Van De Waal D. B. Finke J. F. Visser P. M. Van Donk E. Huisman J. (2014). Rising CO2 levels will intensify phytoplankton blooms in eutrophic and hypertrophic lakes. *PLoS ONE* 9:e104325 10.1371/journal.pone.0104325 PMC4132121 25119996

[B70] Wetzel R. G. (2001). *Limnology - Lake and River Ecosystems.* San Diego, CA: Academic Press.

[B71] Weyhenmeyer G. A. Kosten S. Wallin M. B. Tranvik L. J. Jeppesen E. Roland F. (2015). Significant fraction of CO2 emissions from boreal lakes derived from hydrologic inorganic carbon inputs. *Nat. Geosci.* 8 933–936. 10.1038/ngeo2582

